# A Concise Guide to Silicone-Based Spring-Roll Actuator Assembly

**DOI:** 10.3390/polym15193908

**Published:** 2023-09-27

**Authors:** Gagik Ghazaryan, Alina Khmelnitskaia, Igor Bezsudnov, Aleksandra Kalinina, Elena Agina, Sergey Ponomarenko

**Affiliations:** Enikolopov Institute of Synthetic Polymeric Materials of Russian Academy of Sciences, Profsoyuznaya Str. 70, 117393 Moscow, Russia; gagik@ispm.ru (G.G.); alina.khmelnitskaya@ispm.ru (A.K.); kalinina@ispm.ru (A.K.); agina@ispm.ru (E.A.)

**Keywords:** dielectric elastomers, carbon nanotubes, compliant electrodes, coating, spring-roll actuator

## Abstract

A spring-roll actuator is a multilayer configuration of dielectric elastomer actuators that deforms in response to an electric field. To date, all spring-roll actuators are based on acrylate dielectric elastomers (DEs), and a few can reach deformations on a par with strains observed in natural muscles. Sensitivity to temperature and humidity, as well as the slow response times of acrylates, limit the commercialisation of these actuators. In this work, we developed a spring-roll actuator using commercial silicone DEs because they allow for a broader range of processing temperature and rapid response. Electrodes were deposited on a pre-strained DE film, coated with functional organosilicone polymer composite, and rolled around a metal spring. The coating enhanced the interfacial adhesion between DE and compliant electrodes, preserving the integrity and electro-mechanical properties of the fabricated spring-roll actuator. As to performance, the silicone-based spring-roll actuator could bear 200 times its own weight and displace it by 6% at the applied electric field of 90 V/μm.

## 1. Introduction

**Figure 1:**
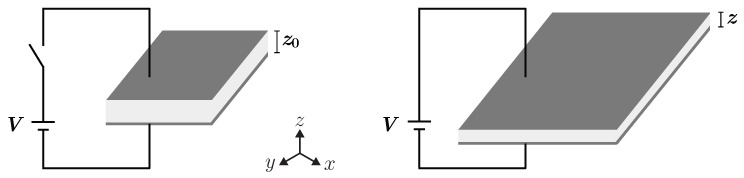
Principle of operation of a DEA.

A dielectric elastomer actuator (DEA) is a capacitor with a thin dielectric elastomer (DE) film sandwiched between two compliant electrodes [1,2]. When an electrical voltage is applied between the electrodes, an electrostatic field occurs and the electrostatic force from the charges on the electrodes mechanically loads the polymer film (Figure 1). Due to this mechanical compression, the (essentially incompressible) elastomer film contracts in the thickness direction. As a result, the polymer material is enlarged elastically in the xy-plane (Maxwell stress). The electrostatic pressure *p* acting on the insulating elastomer film can be calculated for a given applied voltage *V* and film thickness *z* as

(1) $$p=\epsilon_{r}\epsilon_{0}{\left(\frac{V}{z}\right)}^{2},$$

where $\epsilon_{r}$ is the relative permittivity or dielectric constant of the elastomer material, $\epsilon_{0}=8.854\times 10^{-12}\;F/m$ is the vacuum permittivity.

Single-film dielectric elastomers are usually thin, to minimise the actuation voltage. This leads to small force outputs (<10 mN) and limited applications. To reach both the desired forces (>10 N) and displacements (>1 cm), one needs to pack many dielectric and electrode layers.

A multilayer configuration of DEA where the coated DE film is rolled around a spring core is called a “spring-roll” actuator. To increase Maxwell’s electrostatic forces that cause actuation, the DE film may be pre-stretched and the spring may be compressed before wrapping [3,4]. As a result, voltage activation converts the planar expansion of the DE film into linear movement in the axial direction. Thus, the spring-roll actuator, upon utilising basic in-plane expansion, exploits the desirable features of elastomers such as the monolithic structure, flexibility, and multi-functionality.

To date, only a few research groups [5,6,7] have fabricated spring-roll actuators that achieve deformations comparable with the strains observed in natural muscles (20%). These high-performance actuators mostly utilised commercial adhesive films from 3M, the VHB [8] family of acrylic elastomers in particular. VHB acrylates have a relatively high dielectric constant ($\epsilon_{r}=4.7$), elongation (>600%), low moduli at high elongations (0.3–0.6 MPa), and dielectric breakdown strength that may reach >200 V/μm depending on the level of pre-straining [9,10]. Despite their advantages, these spring-roll actuators, and acrylate films in general, have not been successfully commercialised in electrocactive polymer (EAP) devices [11]. There are a few reasons for this. Firstly, acrylates are temperature sensitive: their performance at temperatures outside the range of 0 °C to 70 °C may be 25% lower than the performance at 20 °C, and may even drop to zero by −30 °C [10]. Secondly, an acrylate device may take a long time to reach a stable performance level [12]. Moreover, a study [10] has shown that the performance of an acrylate device increased 25% over the course of a 1 s actuation. Finally, acrylates are sensitive to humidity. The increase in leakage current, which occurs with humidity, decreases the charge-holding capability of the dielectric film and limits its long-term performance. However, a more critical failure mode appears at relatively modest humidity levels. In a recent report [10], the number of cycles to dielectric failure for acrylate devices at different relative humidities at 25 °C was studied. The authors showed that, while no failures were observed after 25,000 cycles at 20% relative humidity, the mean lifetime dropped to 100 cycles above 50% relative humidity.

Silicones have long been studied by EAP researchers [13]. Material properties of silicones are more apt for DE transducer requirements. Electrical resistivity is on the order of $10^{13}$ Ωm and leakage currents can be an order of magnitude lower than those of acrylates. Silicones, like acrylates, are elastic (elongation > 500%) but have a narrower hysteresis loop, modulus ranges from 0.1 to 1 MPa, and show little change in the response with time [10]. Silicones have a broad temperature range with a response that can be constant from −40 to 80 °C. Silicone-based EAP devices are less dependent on environmental conditions and show considerably better long-term reliability performance than acrylate devices [10]. The main drawback of silicone DEs is lower dielectric constant (∼3) compared with acrylates and polyurethanes resulting in lower actuator performance. In the last decade, the number of publications [14,15,16,17,18,19,20,21,22] on the synthesis of novel silicone elastomers with high dielectric constant has risen greatly, nevertheless reports on silicone-based spring-roll actuators remain scarce [23].

In this study, we developed a simple fabrication process for a silicone-based DE wound spring-roll actuator and evaluated its performance. We used a commercial silicone DE film ($\epsilon_{r}=2.8$) for proof of concept, and selected a mixture of carbon nanotubes (CNT) and aminated graphenes (Gr-NH${}_{2}$) as compliant electrodes because they meet the requirement for high conductivity at low thickness [24,25,26,27], and could easily be sprayed onto the DE film using an airbrush [28]. The excellent chemical stability and low surface energy of silicones can result in a poor adhesion with compliant electrodes. To enhance the interfacial adhesion and preserve the integrity and electro-mechanical properties of the fabricated spring-roll actuator, we coated the electrodes with an additional layer of functional organosilicone composition. The coating proved to be advantageous and induced truly compliant electrodes. Finally, a detailed description of the spring-roll fabrication and equipment is presented followed by the estimation of its properties.

## 2. Materials and Methods

### 2.1. Materials

The DE film, a commercial cross-linked silicon film Elastosil^®^ 2030 (50 μm thick), was purchased from Wacker Chemie AG, Germany. Single-walled carbon nanotubes TUBALL^TM^, with an average diameter and length of 1.6±0.4 and 5 μm, respectively, were obtained from OCSiAl, Luxembourg. Aminated graphene (Gr-NH${}_{2}$) was acquired from Graphene Technology, Moscow, Russia. α,ω-hydroxypolydi-methylsiloxane (SKT brand) was purchased from OilGasChemCo Ltd., Moscow, Russia. MQ resin, a copolymer [(CH${}_{3}$)${}_{3}$SiO${}_{0,5}$]${}_{1}$[SiO${}_{2}$]${}_{2}$, was prepared according to the literature [29].

### 2.2. CNT Dispersion Preparation

Prior to dispersing, CNTs were functionalised according to an established procedure [30]. CNTs were oxidised using a 1:2 mixture of H${}_{2}$SO${}_{4}$:HNO${}_{3}$, where the weight ratio of CNTs to the mixture was chosen as 1:50. During the acid treatment, the mixture containing CNTs was vigorously stirred for 1 h followed by ultrasonication for 30 min. The latter was then washed extensively with a deionised water to a neutral pH.

A 0.02 wt% dispersion of functionalised CNTs in ethanol was prepared using a I100-6/4 sonicator at 22 kHz operating frequency for 2 h. After adding aminated graphene to reach a final concentration of 0.12 wt%, the dispersion was ultrasonicated in a water bath for 2 h.

### 2.3. Coating Solution Preparation

The coating solution was made from high-molecular weight polydimethylsiloxane, modified with 3-aminopropyl-diethoxysilyl groups at the chain ends, and MQ-resin prepared according to the literature [29]. It was applied in methyl *tert*-butyl ether at room temperature. The MQ-resin used was a methylsiloxane nanogel with a ratio of M and Q units equal to 1:2, containing residual hydroxyl groups (5.8 wt%).

### 2.4. Spring-Roll Actuator Fabrication

**Figure 2:**
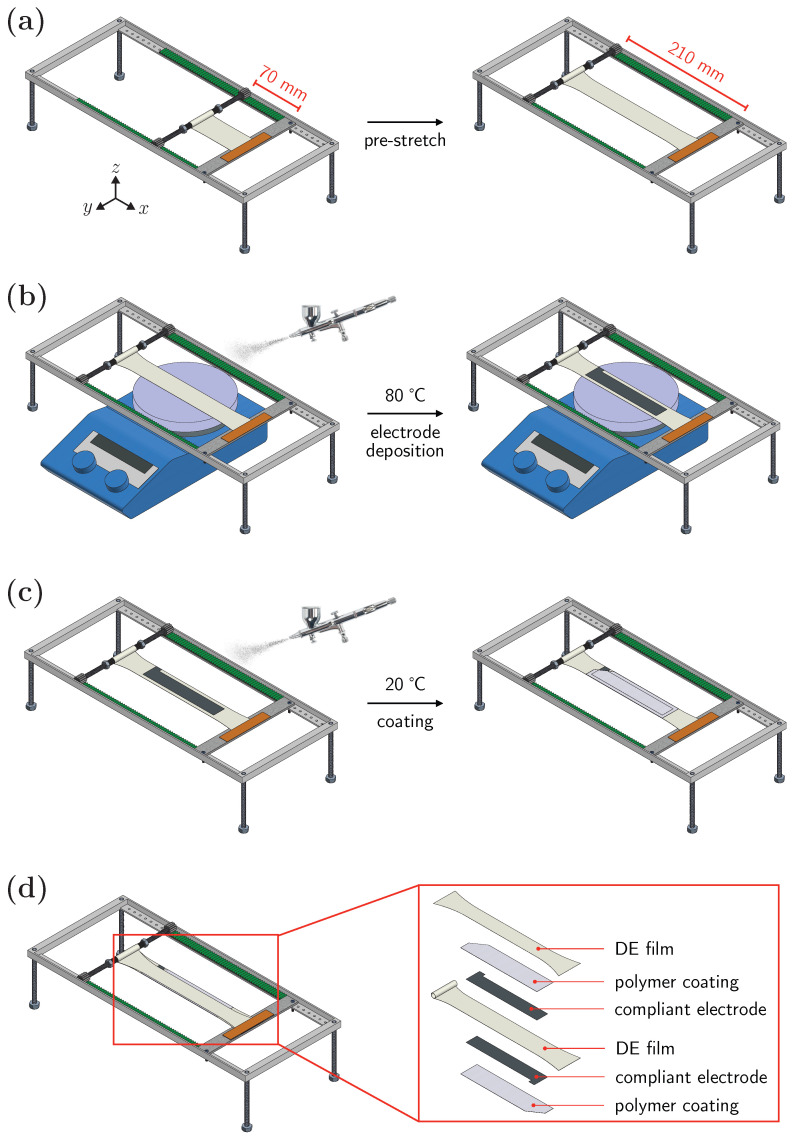
Schematic representation of silicone-based spring-roll actuator fabrication steps: (**a**) pre-stretching, (**b**) electrode deposition, (**c**) coating, and (**d**) the layered structure of DEA setup before winding.

A metal spring that is radially incompressible and axially flexible (with a length and outer diameter of 60 mm and 7 mm, respectively) was slid on an M6×1.0 threaded rod. The spring was compressed to max (55 mm) and held by M6×1.0 nuts. One end of a DE strip, with a size of 100×50 mm${}^{2}$, was wrapped around the spring for two turns, while the other end was fixed in the rigid aluminium frame. The latter was equipped with plastic cog trails, and the threaded rod with plastic cog wheels (Figure 2a). The DE strip was pre-stretched manually to three times its length ($\lambda_{x}^{p}=3$) and fixed. An active electrode area of 135×25 mm${}^{2}$ was isolated using a masking tape, and the DE film was heated to 80 °C on an RCT basic laboratory heater (IKA^®^ Company, Shanghai, China). The CNT/Gr-NH${}_{2}$ dispersion in ethanol was sprayed on each side of the pre-stretched DE film (Figure 2b) using a commercial airbrush (PATRIOT AB 400, China) equipped with an Aspire TC908 compressor (Badger Air-Brush Co., Bellwood, IL, USA). The spraying was peformed at ca. 50 psi pressure using a nozzle with 0.3 mm diameter. The samples prepared in this manner are referred to as uncoated DEA. For coated DEA, the coating solution was sprayed over the electrodes at room temperature with a similar airbrush (Figure 2c). The samples were left in a fume hood for the solvent to evaporate for at least an hour. A pristine DE strip was fixed on top of the pre-stretched DEA prior to the rolling. The layered structure of the system is highlighted in Figure 2d. The DEA was wrapped around the spring as the threaded rod carrying the spring was manually rolled towards the edge. Copper wires were attached to the compliant electrodes during the rolling. All in all, the two DE films were rolled around the spring 7 turns, 2 of which (1 turn at the beginning and 1 turn at the end) carried no compliant electrodes. Thus, only 10 layers out of the 14-layer silicone-based spring-roll actuator could respond to an applied electric field.

### 2.5. Adhesion Test

The adhesion between the DE film and the compliant electrodes was measured qualitatively via the Scotch^®^ tape test. CNT/Gr-NH${}_{2}$ electrodes were deposited on a DE surface area of 110×10 mm${}^{2}$. One half of it was coated with functional organosilicone composition, while the other half was left uncoated. A transparent Scotch^®^ tape (20 mm wide) was pressed against the surface containing the electrodes. The adhesive tape was removed manually, and the amount of the electrodes attached to its surface was examined visually.

### 2.6. Raman Spectroscopy

The Raman measurements were performed using a Raman microscope (inVia, Renishaw plc, Spectroscopy Product Division, Old Town, Wotton-Under-Edge, Gloucestershire, GL12 7DW, UK) with a 50× objective lens (Leica DM 2500 M, NA =0.75, Leica Mikrosysteme Vertrieb GmbH Mikroskopie und Histologie Ernst-Leitz-Strasse 17–37 Wetzlar, 35578 Germany). The measurements were made with an edge filter, the excitation wavelength was 633 nm, as provided by an He–Ne laser (RL633, Renishaw plc, Spectroscopy Product Division, Old Town, Wotton-Under-Edge, Gloucestershire, GL12 7DW, UK) with a maximum power of 17 mW. The acquisition time and number of accumulations were adjusted to maximise the signal-to-noise ratio with the minimal sample degradation. All the spectra for the samples were measured at several points and then averaged to reduce the anisotropy effect on the Raman spectra and to increase the single-to-noise ratio. The background from the Raman spectra was subtracted by the cubic spline interpolation method. All the spectra were divided by the number of accumulations and the acquisition time.

### 2.7. Scanning Electron Microscopy (SEM)

A JCM-6000Plus versatile benchtop SEM (Jeol, Tokyo, Japan) was used to obtain cross-sectional images. Two types of samples were prepared for SEM imaging—uncoated and coated DEAs. The samples were sandwiched between two rectangular sample holders made out of polyethylene (size of 10×10 mm${}^{2}$), which were mounted in the microtome, and cut with a razor blade at 20 °C. A thin layer of gold was sputtered on the cross-sectional cut using a 29030SCTR Smart Coater (Jeol, Japan).

### 2.8. Tensile Test

Mechanical properties of uncoated and coated DEAs were measured on a Mecmesin static tensile tester (Mecmesin Ltd., Slinfold, West Sussex, UK) at 20 °C. The samples were uniaxially stretched at a drawing speed of 0.1 s^−1^, and the values of drawing force and deformation were recorded. The latter were converted into engineering stress and strain and are presented as stress–strain curves.

### 2.9. Capacitance Measurements

Capacitance of the DE membrane module was measured on a digital multimeter MNIPI E7-20 (DEOMERA, Cheboksary, Russia) at 20 °C. The uncoated and coated DEAs were uniaxially stretched on a Mecmesin static tensile tester, and the absolute capacitance (*C*) values were recorded at different draw ratios ($\lambda_{x}$). The drawing was performed at a speed of 1 s^−1^. The capacitance of the spring-roll actuator was measured before and after hanging a 1 kg load.

### 2.10. Dielectric Breakdown Test

For dielectric breakdown test five samples of DEAs, coated and uncoated, were fabricated and measured on a GPT-79803 compliance tester (GW Instek, New Taipei City, Taiwan). The breakdown test was realised as a progressive stress test with a linear increase of the electrical voltage (direct current) from zero until breakdown occurred. The voltage slew rate was 200 V/s. The nominal dielectric breakdown field strength $E_{BRnom}$ was calculated by dividing the measured breakdown voltage $V_{BR}$ by the initial thickness of the DE film $z_{0}$:

(2) $$E_{BRnom}=\frac{V_{BR}}{z_{0}}.$$

### 2.11. Actuation Experiment

The mechanical response of the spring-roll actuator was measured on a GPT-79803 compliance tester (GW Instek, Taiwan). The spring-roll actuator was fixed vertically on one end, and a load of 1 kg was hung from the other one. The spring-roll actuator was activated with increasing the activation voltage (direct current) from 0 to 2 kV. Breakdown tests showed that the actuators with DE film thickness of approx. 22 μm became electromechanically unstable over an activation voltage of 2.2 kV. To avoid electrical breakdown the activation voltage was therefore limited to 2.0 kV. The tests were carried out gradually with 300 V steps up to 1.5 kV and subsequently with 100 V steps up to 2.0 kV. The corresponding displacement was measured after a short rest of 3 s, waiting for a stable situation in each case. The voltage ON/OFF cycle was repeated 10 times.

## 3. Results

**Figure 3:**
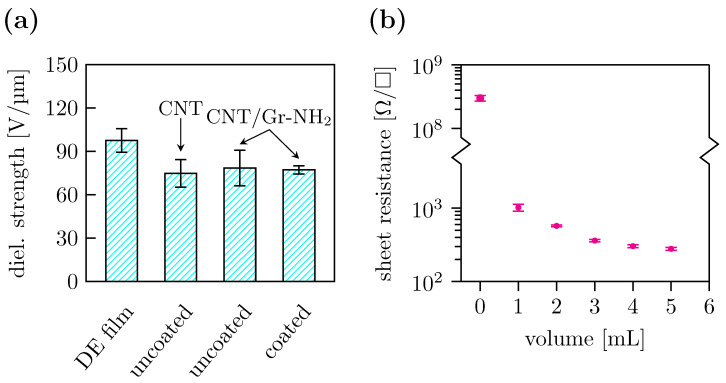
(**a**) Dielectric breakdown performance of the DEAs with different compliant electrodes. (**b**) The sheet resistance of CNT/Gr-NH2 electrodes deposited on pre-stretched DE film as a function of dispersion volume sprayed. The error bars correspond to the standard deviation of the dataset.

To test the effect of coating on the breakdown field, we fabricated DEAs using CNT/Gr-NH${}_{2}$ electrodes—uncoated and coated. We also prepared a DEA using CNT electrodes exclusively for comparison. The dielectric breakdown field was measured in each sample and presented in Figure 3a. Pristine DE membrane showed the highest breakdown field, 98±8 V/μm. Amongst the uncoated DEAs, the samples carrying CNT/Gr-NH${}_{2}$ electrodes showed a slightly higher breakdown field than those with CNT electrodes (79±12 V/μm vs. parse-numbers=false 75±9 V/μm). Finally, the breakdown field of coated DEAs appeared in the same range as uncoated samples, 77±3 V/μm.

We selected CNT/Gr-NH${}_{2}$ as compliant electrodes because (i) these DEAs had higher breakdown fields compared with the CNT electrodes, and (ii) the CNT/Gr-NH${}_{2}$ dispersion in ethanol was more stable and contained less bundles than the CNT dispersion of the same concentration. This is due to strong interactions between carboxylic acid groups, generated on CNT surfaces through acid treatment, and amino groups of aminated graphene [26]. As a result, the spraying proceeds easily and smoothly—without clogging the nozzle.

To optimise the amount of CNT/Gr-NH${}_{2}$ electrodes in a DEA, we calibrated the process by measuring the sheet resistance of the electrodes at various volumes of dispersion sprayed (Figure 3b). The sheet resistance of the pristine DE film was 2 × 10^8^ Ω/□. It dropped by a factor of $10^{5}$ after spraying 1 mL dispersion onto its surface. The sheet resistance further decreased upon spraying more dispersion and levelled off at 5 mL. A 5 mL dispersion appeared to be the optimum amount for depositing a layer of compliant electrode.

**Figure 4:**
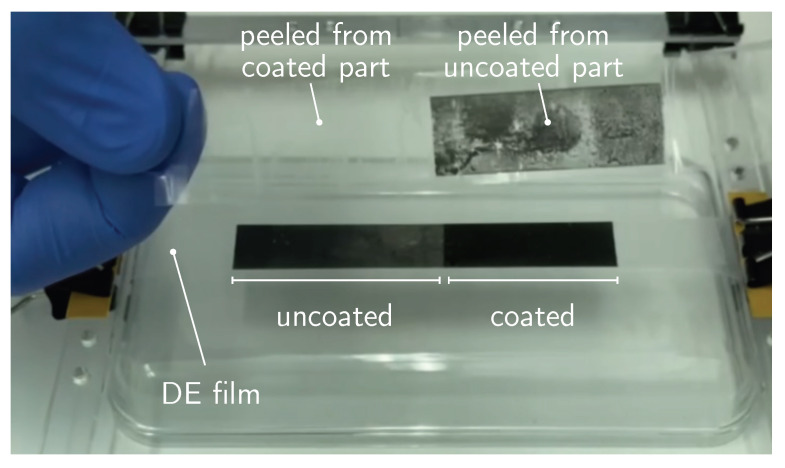
Peel test demonstrating the adhesive properties of the electrodes.

The integrity of DEA depends strongly upon the interfacial adhesion between the DE film and the compliant electrodes. Hence, the interfacial adhesion of the compliant electrodes in uncoated and coated DEAs were evaluated qualitatively as follows: a layer of CNT/Gr-NH${}_{2}$ was deposited on a surface of pre-stretched DE film, approximately half of which was coated. A commercial Scotch^®^ tape was adhered to the surface, firmly pressed against it, and peeled off as shown in Figure 4 and in the Supporting Information. A freeze-frame image of Movie S1 highlights the two areas of the peeled Scotch^®^ tape. While the area corresponding to the uncoated surface is entirely covered with CNT/Gr-NH${}_{2}$ electrodes, the coated surface remains intact. The peel test demonstrates how strongly the electrodes are adhered to the DE surface in coated DEA in contrast to the uncoated moieties.

**Figure 5:**
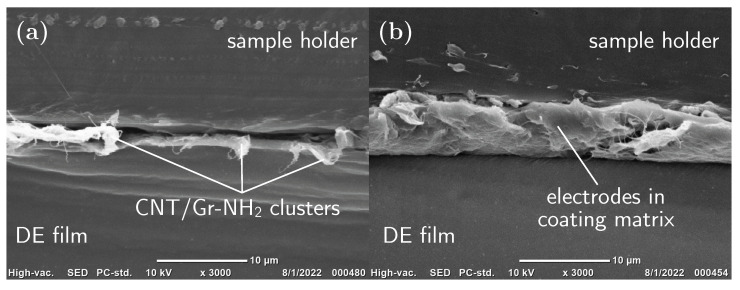
Cross-sectional SEM images of the interface between DE film and electrode layer. (**a**) CNT/Gr-NH${}_{2}$ form clusters at the interface in the uncoated specimen. (**b**) Coating embeds the electrodes forming a matrix with a good interfacial adhesion within the matrix as well as between polymer coating and DE film.

SEM imaging of a specimen cross-section was carried out to observe the interface between the DE and the compliant electrodes. Typical SEM cross-sectional images of uncoated and coated samples are presented in Figure 5. Because of a poor interfacial adhesion between the uncoated DE film and compliant electrodes, the razor blade distorted the electrode layer during the sample preparation. As a result, randomly located clusters of CNT/Gr-NH${}_{2}$ can be seen at the interface (Figure 5a). The presence of the electrodes (CNT and Gr-NH${}_{2}$) was also confirmed by Raman spectroscopy (Figure S1, Supplementary Information). Coating preserved the integrity of the electrodes by forming a matrix comprising CNT/Gr-NH${}_{2}$ and coated functional organosilicone polymer composite (Figure 5b). The interfacial adhesion in this matrix as well as between DE film and the coated polymer composite is good enough to maintain the DEA undamaged for practical uses, sample preparation, handling, etc. In addition, SEM imaging provided information on the layer thickness in uncoated and coated electrodes. The average thickness of uncoated electrodes, estimated from undistorted regions, was ca. 2 μm. The average thickness of coated electrodes reached 8 μm.

Raman scattering further proved that the compliant electrodes consist of a mixture of CNT/Gr-NH${}_{2}$, and the coating is an organosilicone polymer composite similar to the DE film. The Raman spectrum of the compliant electrodes (Figure S1a) showed three band peaks, including a D-band (1342 cm^−1^), a G-band (1573 cm^−1^), and a 2D-band (2690 cm^−1^) characteristic to both CNTs and graphene. However, the strong 2D band of CNT/Gr-NH${}_{2}$ may be attributed to the presence of aminated graphene in the electrodes [31]. The Raman spectrum of the coated DE film showed some characteristic band peaks at 489, 709 and 2906 cm^−1^ corresponding to symmetric Si-O, asymmetric C-Si-C and symmetric C-H stretching, respectively [32]. In addition, the Raman imaging confirms the presence of the uniform organosilicone coating layer (Figure S1b,c).

**Figure 6:**
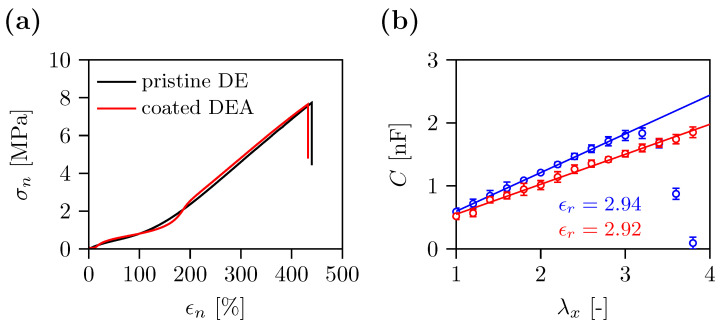
(**a**) Engineering stress–strain curves of uniaxially drawn pristine DE film and coated DEA. (**b**) Capacitance (*C*) of uncoated (○) and coated (○) DEAs as a function of uniaxial drawing ($\lambda_{x}$). Dielectric constants ($\epsilon_{r}$) were calculated from the fitted curves (blue and red solid lines) according to Equation (4). The error bars correspond to the standard deviation of the data set.

Having established the procedure for depositing compliant electrodes followed by functional organosilicone polymer composite coating, we were interested in testing the mechanical properties of coated DEA. Engineering stress–strain curves of the latter are compared to the pristine DE film in Figure 6a. The two curves match well with characteristic parameters such as Young’s modulus, strain hardening, curve shape, strain at break and ultimate strength. For example, both curves display a strain of break at ca. 430%, a Young’s modulus of 1 MPa estimated at 0–10% and a strain hardening modulus of 2.2 MPa at 200–400%.

Prior to spring-roll actuator fabrication, the capacitance of DEAs was examined. In theory, capacitance (*C*) of a DE film sandwiched between compliant electrodes can be calculated as:

(3) $$C=\epsilon_{r}\epsilon_{0}\frac{A}{z},$$

where $\epsilon_{r}$ is the relative permittivity or dielectric constant of the elastomer material, $\epsilon_{0}=8.854\times 10^{-12}\;F/m$ is the vacuum permittivity, *A* is the active electrode area, and *z* is DE film thickness. Assuming that the uniaxial deformation of the DE film is volume preserving ($\lambda_{x}\lambda_{y}\lambda_{z}=1$) [33], we can rearrange Equation (3) into

(4) $$C=\frac{\epsilon_{r}\epsilon_{0}A_{0}}{z_{0}}\lambda_{x},$$

where $A_{0}$ is the active electrode area of unstretched DE film, $z_{0}$ is the initial thickness of the DE film, $\lambda_{x}$ is the draw ratio in the *x*-direction. The capacitance was measured while the DEAs were drawn uniaxially to a draw ratio $\lambda_{x}=4$. The plot of capacitance as a function of the drawing ratio is presented in Figure 6b. For uncoated DEA, the capacitance linearly increased from 0.6 nF to the highest 1.8 nF then declined abruptly almost to zero. The capacitance of coated DEA increased linearly from 0.5 nF to 1.9 nF at the entire drawing range. The linear increase in the capacitance was evaluated by fitting Equation (4) to the data at a drawing range of 1 to 3 and 1 to 4 for uncoated and coated DEAs, respectively. We calculated the dielectric constant of the DE film from the slope of the fitted curves. The obtained values, $\epsilon_{r}=2.94$ and $\epsilon_{r}=2.92$, match well the value reported in the literature [34]. The capacitance of uncoated DEAs are slightly higher than the values of coated samples. This could be ascribed to the larger thickness of coated samples compared with uncoated DEA.

**Figure 7:**
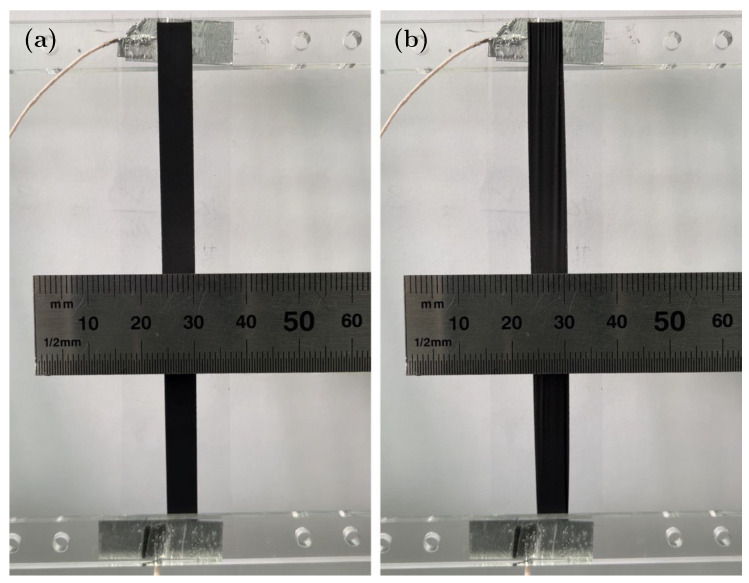
Mono-laye actuator before (**a**) and after (**b**) applying electrical power. The actuator at 3 kV showed a 1 mm displacement in the *y*-direction.

Prior to testing the spring-roll actuator, we prepared a mono-layer actuator and measured the actuation strain of the active electrode stripe in the *y*-direction. The mono-layer actuator was pre-strained uniaxially in the *x*-direction 2×, fixed, and exposed to gradually increasing electric field. At 3 kV applied voltage, which translates into ca. 100 V/μm, a 1 mm displacement was recorded (Figure 7, Movie S2). The latter corresponds to an actuation strain of 17% in the *y*-direction.

**Figure 8:**
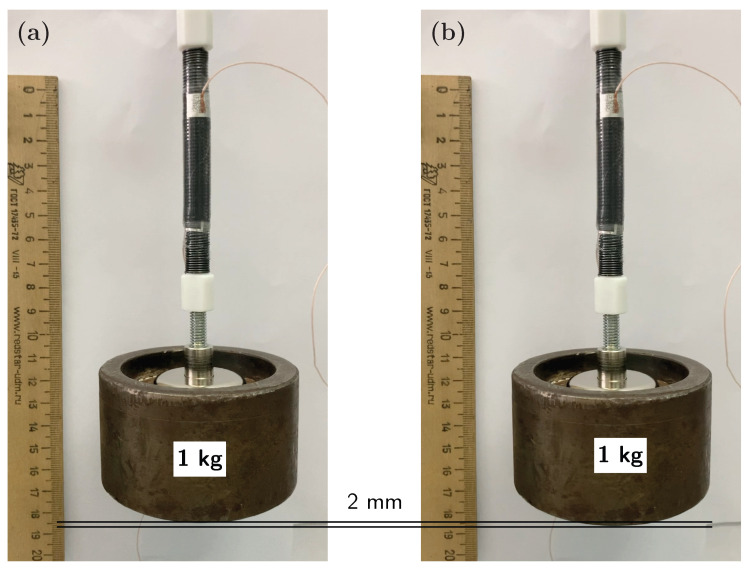
Spring-roll actuator attached to a 1 kg weight before (**a**) and after (**b**) applying electrical power. The actuator is 52 mm long and weighs 5 g. At 2 kV the spring-roll displaced the hanging weight by 2 mm.

The performance of the spring-roll actuator as an artificial muscle was evaluated by measuring the free displacement of the loaded actuator at the applied electric field (Figure 8, Movie S3). One end of the spring-roll actuator was held fixed and a 1 kg hanging weight was attached to the other end. At this point, the DE film had gone through two types of deformation: uniaxial stretching in the *x*-direction during the fabrication ($\lambda_{x}=3$), then planar extension in the *y*-direction when a 1 kg was hung ($\lambda_{y}=1.3$). This reduced the thickness of the DE film from 50 μm to 22 μm (in the *z*-direction). At the applied voltage of 2 kV, which translates into 90 V/μm electric field, the hanging weight was vertically displaced by 2 mm due to the expansion of the spring-roll actuator in the *y*-direction. The latter corresponds to an actuation strain of 6%. The actuation experiment was repeated for 10 cycles (Movie S4), and identical results were obtained in each cycle.

**Figure 9:**
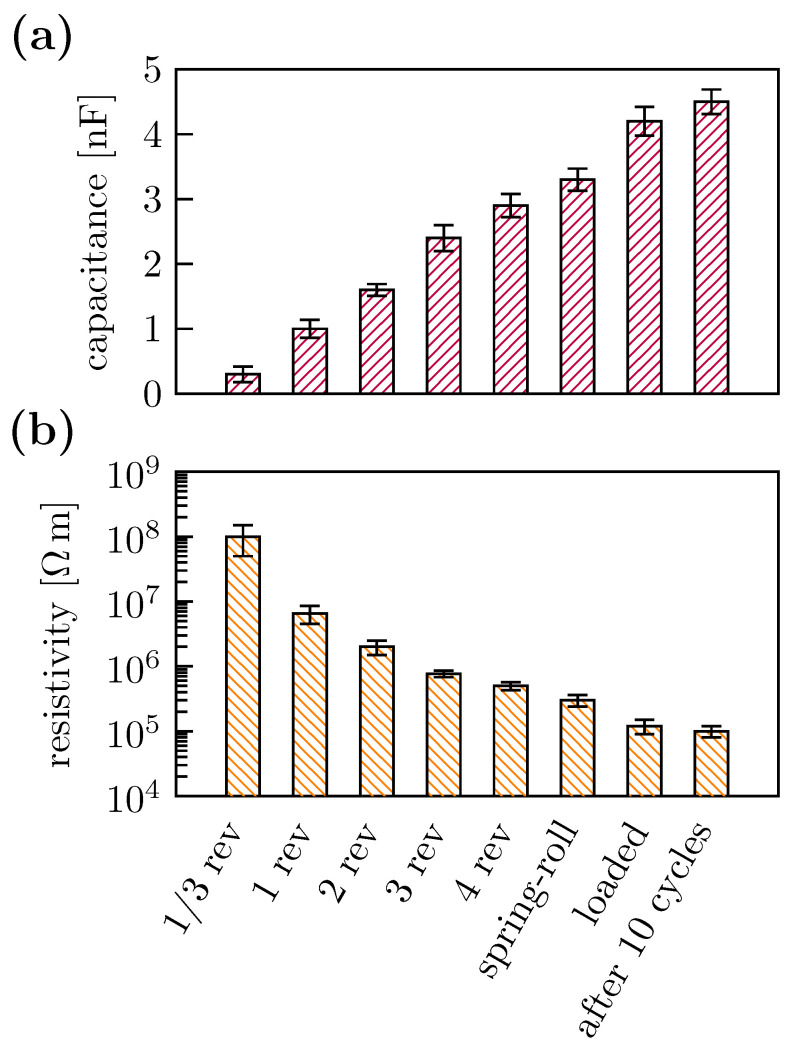
Monitoring the capacitance (**a**) and the volume resistivity (**b**) of the spring-roll actuator during the fabrication.

During the spring-roll fabrication, we kept monitoring two characteristic parameters of the actuator: the capacitance and the volume resistivity of the DE film. Inspection of the capacitance (Figure 9a) of the spring-roll actuator showed that it increased from 0.3 nF to 3 nF between the first and the forth (last) revolutions, reaching to 4.2 nF and 4.5 nF for the spring-roll actuator loaded with 1 kg weight before and after 10 cycles of actuation, respectively. The volume resistivity, on the contrary, dropped tremendously (Figure 9b). The resistivity of the mono-layer actuator film before wrapping around the spring was roughly 10^8^ Ωm. It exponentially decreased to $1.2\times {\mathrm{10}}^{5}$ Ωm for the spring-roll actuator loaded with 1 kg weight before the applied voltage, eventually reaching ${\mathrm{10}}^{5}$ Ω. m after 10 cycles.

## 4. Discussion

We have developed a robust method for silicone-based spring-roll actuator fabrication using a commercial silicone film as DE. We chose a dispersion of CNT/Gr-NH${}_{2}$ in ethanol for spraying compliant electrodes, then coated the electrodes with functional organosilicone polymer composite prepared as reported earlier [29]. Aminated graphene (i) stabilises the CNT dispersion, (ii) facilitates the spraying and (iii) creates a uniform layer of compliant electrodes with an average thickness of 2 μm. The coating, as observed by cross-sectional SEM imaging (Figure 5) and Raman spectroscopy (Figure S1), is a matrix where the coated polymer composite embeds the CNT/Gr-NH${}_{2}$ electrodes. A Scotch^®^ tape peel test revealed that coating increases the interfacial adhesion between DE film and resulted matrix (Figure 4). On the one hand, hydrogen bonds between −NH${}_{2}$ groups of aminated graphene, −OH and −COOH groups of CNTs and the residual hydroxyl groups of MQ-resin in the coating polymer result in a good adhesion within the matrix. On the other hand, hydrophobic interactions between DE film and coated polymer originate a strong interfacial adhesion. Consequently, a layer of truly compliant electrodes are created on a pre-stretched DE film that remains intact for practical uses and handling.

We evaluated the effect of electrode deposition and coating on the electro-mechanical properties of DEAs. The results of the tensile testing shown in Figure 6 revealed that the mechanical properties of the coated DE film actuator were fully preserved. In other words, neither the stiffness nor the flexibility of the DE were affected by the electrode deposition and/or coating. Breakdown field measurements revealed that the dielectric breakdown of pristine DE film was affected during the sample fabrication (Figure 3). Particularly, dielectric breakdown of all DEAs, regardless of the electrode type and coating, decreased with respect to pristine DE film. A similar effect was published in a recent paper [35], where the authors investigated the relationship between the areal density of CNTs and the onset of dielectric breakdown. We believe that the reason for early dielectric breakdown is the inhomogenous distribution of electrodes causing high electric field concentrations near to sharp edges or needles of CNT/Gr-NH${}_{2}$ laying on the membrane surface. This is relevant to our samples because the electric conductors in our electrodes are CNTs, and the coating has no effect on the dielectric breakdown (Figure 3).

Capacitance measurements of DEAs showed a negligible difference between the coated and uncoated samples in terms of slope and absolute values (Figure 6b). But they revealed the main advantage of the coating—the samples could be stretched in the *x*-direction further than the pre-straining ($\lambda_{x}^{p}=3$) without affecting the electro-mechanical properties of the DEA. Furthermore, the electro-mechanical properties remained preserved in the fabricated spring-roll actuator, where the DEA was stretched in the *y*-direction.

In an actuation experiment (see Supporting Information), the mono-layer actuator extended in the *y*-direction by 17%. In theory, the latter can be calculated from the thickness strain, which can be estimated using the model proposed by Pelrine et al. [13]. According to the model, the thickness strain ($s_{z})$ can be written as

(5) $$s_{z}=-\frac{\epsilon_{r}\epsilon_{0}}{Y}{\left(\frac{V}{z}\right)}^{2},$$

where $\epsilon_{r}$ is the relative permittivity or dielectric constant of the elastomer material, $\epsilon_{0}=8.854\times 10^{-12}\;F/m$ is the vacuum permittivity, *Y* is the Young’s modulus of the DE, *V* is the applied voltage, *z* is the thickness. The actual dimensions of the film can be found from the strains and its initial dimensions. For example, with the thickness we have $z=z_{0}\left(s_{z}+1\right)$, where $z_{0}$ is the initial thickness. In our experiment, the actuator is constrained in the *x*-direction (i.e., $s_{x}=0$), therefore the condition of the constant volume $\left(s_{z}+1\right)\left(s_{y}+1\right)\left(s_{x}+1\right)=1$ may be rewritten as $\left(s_{z}+1\right)\left(s_{y}+1\right)=1$. It follows directly from the last expression that the actuation strain in the *y*-direction ($s_{y}$) can be presented as

(6) $$s_{y}=\frac{1}{s_{z}+1}-1.$$

We calculated the actuation strain ($s_{y}$) using Equations (5) and (6) under the experimental conditions and given the materials characteristic parameters. We found a good agreement between theoretical (16%) and measured (17%) actuation strains at the tested voltage.

In an actuation experiment (see Supporting Information) the spring-roll actuator contracted a 1 kg hanging weight by 2 mm at 90 V/μm applied electric field. The actuator consists of 14 layers: 10 active layers and 4 layers for mechanical support; and weighs 5 g. For load-bearing, it can support 200× its own weight and displace it vertically by 6%. While the former exceeds by far the existing spring-roll [5,7,36,37], core-free [38,39,40,41,42] or stacked actuators [35,43], the latter falls short of acrylate-based spring-roll actuators.

The most controversial result is that the mono-layer actuator produces almost 3× more actuation strain than the spring-roll actuator despite the latter has much higher capacitance. A study reported a comparison of the flat and rolled actuator configuration yielding in a greater displacement of 20% for the flat configuration than the rolled actuator (6%) at an applied field of 50 V/μm [39]. The authors attributed this difference to the manual fabrication techniques used to construct the rolled actuator. In particular, the electric field may not be applied perpendicularly across the film thickness because the patterned electrodes were not perfectly aligned during the fabrication process.

The existing models on spring-roll actuators [7,38], in fact, suggest that the spring-roll actuators should produce higher actuation strains than their mono-layer counterparts at the same electric field. In the model developed by Rajamani et al. [7], the thickness strain can be expressed as

(7) $$s_{z}=-\frac{\sqrt{2}\epsilon_{r}\epsilon_{0}}{Y}{\left(\frac{V}{z}\right)}^{2}.$$

**Figure 10:**
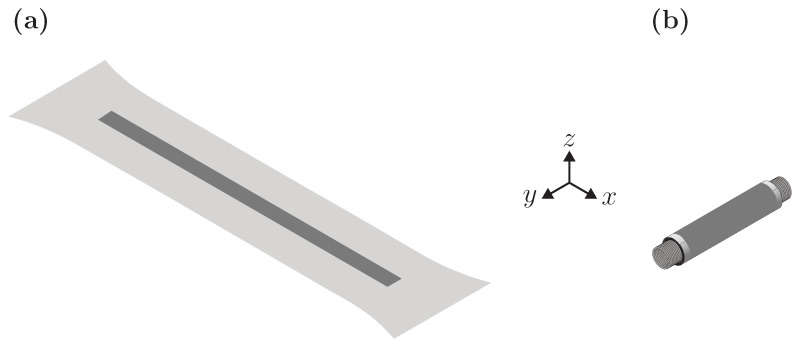
A scheme illustrating the type of deformation for mono-layer DEA (**a**) and spring-roll actuator (**b**). In both cases, the DE film is fixed in the *x*-direction, therefore it can only undergo a planar extension in the *y*-direction when a voltage is applied.

This implies that the thickness strain must be higher by a factor of $\sqrt{2}$. Since the spring-roll and the mono-layer actuators undergo the same deformation (Figure 10), the actuation strain in the *y*-direction must be higher for the former (about 22% according to Equations (6) and (7)).

The discrepancy between theoretical (22%) and experimentally observed (6%) axial strains may be explained by examining the change in the volume resistivity during the spring-roll fabrication (Figure 9). The resistivity of a functional spring-roll actuator is roughly 10^5^ Ωm—three orders of magnitude lower than its mono-layer counterpart. This reduces the energy conversion efficiency of the spring-roll actuator, resulting in a significantly lower axial strain. We hypothesise that randomly distributed individual CNTs penetrated into the DE film when it wrapped around the spring. Wrapping presses the layers tightly causing the CNTs to “pierce” the DE film. This, along with the friction between the DE and the CNT layers, can micro-damage the DE film and, therefore, reduce the resistivity.

## 5. Conclusions

We have developed a simple and robust method for silicone-based spring-roll actuator fabrication using a mixture of CNTs and aminated graphene as compliant electrodes. The key step to the fabrication process—the coating of the electrodes with functional organosilicone polymer composite—enhances the interfacial adhesion between the DE film and the electrodes, creating a layer of truly compliant electrodes that remains intact for practical uses and handling. Meanwhile, the mechanical and electrical properties of the spring-roll actuator are fully preserved.

While the mono-layer actuator showed 17% actuation strain, the spring-roll actuator could support 200× its own weight and displace it by 6%. The declining axial strain is a result of reducing the volume resistivity of the DE membrane. We hypothesise that during the spring-roll fabrication, the electrodes are pressed against the DE film causing a formation of micro-cracks.

We compared our work with rolled DEAs reported earlier (Table S1, Supporting Information). While our silicone-based spring-roll exceeded the rolled DEAs or even the acrylate-based spring-roll actuators by a number of parameters, it fell short in terms of the other parameters. In the future, we propose two strategies to improve our work. Firstly, we think that the coating of CNT electrodes with a functional organosilicone polymer composite from both sides would isolate the CNT layer from the DE film and would prevent micro-crack formation. Secondly, an alternative method for CNT deposition—dry film transfer technology developed by Nasibulin and co-workers [44,45]—should overcome this problem mainly because the CNT films are in the range of 50 nm, flat, and lack randomly distributed “sharp” ends that can damage the DE film.

## Data Availability

Data available on request.

## Supplementary Materials

The following supporting information can be downloaded at: https://www.mdpi.com/article/10.3390/polym15193908/s1, Figure S1: (a) Raman spectra of pristine DE film (green), DE film with the CNT/Gr-NH${}_{2}$ deposited on it (red) and DE film with the CNT/Gr-NH${}_{2}$ coated with the functional organosilicone polymer composite (blue). The images of the latter two are shown in (b,c), respectively. Description is presented in the text in detail; Table S1: Comparison among various rolled DEAs reported so far; Supplementary Videos S1–S4 associated with this article can be found in the online version.

## References

[1] Bar-Cohen Y., Anderson I.A. (2019). Electroactive polymer (EAP) actuators—Background review. Mech. Soft Mater..

[2] Cao X., Zhang M., Zhang Z., Xu Y., Xiao Y., Li T. (2019). Review of Soft Linear Actuator and the Design of a Dielectric Elastomer Linear Actuator. Acta Mech. Solida Sin..

[3] Youn J.H., Jeong S.M., Hwang G., Kim H., Hyeon K., Park J., Kyung K.U. (2020). Dielectric Elastomer Actuator for Soft Robotics Applications and Challenges. Appl. Sci..

[4] Hajiesmaili E., Clarke D.R. (2021). Dielectric elastomer actuators. J. Appl. Phys..

[5] Pei Q., Pelrine R., Stanford S., Kornbluh R., Rosenthal M. (2003). Electroelastomer rolls and their application for biomimetic walking robots. Synth. Met..

[6] Kovacs G., Lochmatter P., Wissler M. (2007). Arm-wrestling robot driven by dielectric elastomer actuators. Smart Matter. Struct..

[7] Rajamani A., Grissom M.D., Rahn C.D., Zhang Q. (2008). Wound Roll Dielectric Elastomer Actuators: Fabrication, Analysis, and Experiments. IEEE ASME Trans. Mechatron..

[8] 3MTM VHBTM Tapes. https://www.3m.com/3M/en_US/vhb-tapes-us/.

[9] Kofod G., Sommer-Larsen P., Kornbluh R., Pelrine R. (2003). Actuation response of polyacrylate dielectric elastomers. J. Intell. Mater. Syst. Struct..

[10] Biggs J., Danielmeier K., Hitzbleck J., Krause J., Kridl T., Nowak S., Orselli E., Quan X., Schapeler D., Sutherland W. (2013). Electroactive polymers: Developments of and perspectives for dielectric elastomers. Angew. Chem. Int. Ed..

[11] Bezsudnov I., Khmelnitskaia A., Kalinina A., Ponomarenko S. (2023). Materials and design of dielectric actuators. Russ. Chem. Rev..

[12] Plante J.S., Dubowsky S. (2007). On the properties of dielectric elastomer actuators and their design implications. Smart Mater. Struct..

[13] Pelrine R., Kornbluh R., Joseph J. (1998). Electrostriction of polymer dielectrics with compliant electrodes as a means of actuation. Sens. Actuators A.

[14] Dünki S.J., Ko Y.S., Nüesch F.A., Opris D.M. (2015). Self-Repairable, High Permittivity Dielectric Elastomers with Large Actuation Strains at Low Electric Fields. Adv. Func. Mater..

[15] Dünki S.J., Nüesch F.A., Opris D.M. (2016). Elastomers with tunable dielectric and electromechanical properties. J. Mater. Chem. C.

[16] Caspari P., Dünki S.J., Nüesch F.A., Opris D.M. (2018). Dielectric elastomer actuators with increased dielectric permittivity and low leakage current capable of suppressing electromechanical instability. J. Mater. Chem. C.

[17] Perju E., Cuervo-Reyes E., Shova S., Opris D.M. (2018). Synthesis of novel cyclosiloxane monomers containing push-pull moieties and their anionic ring opening polymerization. RCS Adv..

[18] Caspari P., Nüesch F.A., Opris D.M. (2019). Synthesis of solvent-free processable and on-demand cross-linkable dielectric elastomers for actuators. J. Mater. Chem. C.

[19] Sheima Y., Caspari P., Opris D.M. (2019). Artificial Muscles: Dielectric Elastomers Responsive to Low Voltages. Macromol. Rapid Commun..

[20] Perju E., Ko Y.S., Dünki S.J., Opris D.M. (2020). Increased electromechanical sensitivity of polysiloxane elastomers by chemical modification with thioacetic groups. Mater. Des..

[21] Perju E., Shova S., Opris D.M. (2020). Electrically Driven Artificial Muscles Using Novel Polysiloxane Elastomers Modified with Nitroaniline Push-Pull Moieties. ACS Appl. Mater. Interfaces.

[22] Sheima Y., Yuts Y., Frauenrath H., Opris D.M. (2021). Polysiloxanes Modified with Different Types and Contents of Polar Groups: Synthesis, Structure, and Thermal and Dielectric Properties. Macromolecules.

[23] Zhang X., Löwe C., Wissler M., Jähne B., Kovacs G. (2005). Dielectric elastomers in actuator technology. Adv. Eng. Mater..

[24] Rosset S., Shea H.R. (2013). Flexible and stretchable electrodes for dielectric elastomer actuators. Appl. Phys. A.

[25] Shian S., Diebold R.M., McNamara A., Clarke D.R. (2012). Highly compliant transparent electrodes. Appl. Phys. Lett..

[26] Chae S.H., Lee H.Y. (2014). Carbon nanotubes and graphene towards soft electronics. Nano Converg..

[27] Lv T.R., Zhang W.H., Yang Y.Q., Zhang J.C., Yin M.J., Yin Z., Yong K.T., An Q.F. (2023). Micro/Nano-Fabrication of Flexible Poly(3,4-Ethylenedioxythiophene)-Based Conductive Films for High-Performance Microdevices. Small.

[28] Kaempgen M., Duesberg G.S., Roth S. (2005). Transparent carbon nanotube coatings. Appl. Surf. Sci..

[29] Meshkov I.B., Kalinina A.A., Gorodov V.V., Bakirov A.V., Krasheninnikov S.V., Chvalun S.N., Muzafarov A.M. (2021). New principles of polymer composite preparation. MQ copolymers as an active molecular filler for polydimethylsiloxane rubbers. Polymers.

[30] Porto A.B., Silva G.G., Santos H.F.D., de Oliveira L.F. (2018). Oxidation of single-walled carbon nanotubes under controlled chemical conditions. J. Braz. Chem. Soc..

[31] Feng J.M., Dai Y.J. (2013). Water-assisted growth of graphene on carbon nanotubes by the chemical vapor deposition method. Nanoscale.

[32] Jayes L., Hard A., Séné C., Parker S., Jayasooriya U. (2003). Vibrational Spectroscopic Analysis of Silicones: A Fourier Transform-Raman and Inelastic Neutron Scattering Investigation. Anal. Chem..

[33] Koh S.J.A., Li T., Zhou J., Zhao X., Hong W., Zhu J., Suo Z. (2011). Mechanisms of large actuation strain in dielectric elastomers. J. Polym. Sci. Part B Polym. Phys..

[34] Förster-Zügel F., Solano-Arana S., Klug F., Schlaak H.F. (2019). Dielectric breakdown strength measurements with silicone-based single-layer dielectric elastomer transducers. Smart Mater. Struct..

[35] Duduta M., Hajiesmaili E., Zhao H., Wood R.J., Clarke D.R. (2019). Realizing the potential of dielectric elastomer artificial muscles. Proc. Natl. Acad. Sci. USA.

[36] Kovacs G., Ha S.M., Michel S., Pelrine R., Pei Q. (2008). Study on core free rolled actuator based on soft dielectric EAP. Proc. SPIE.

[37] Zhang R., Lochmatter P., Kunze A., Gabor G. (2006). Spring roll dielectric elastomer actuators for a portable force feedback glove. Proc. SPIE.

[38] Carpi F., De Rossi D. (2004). Dielectric elastomer cylindrical actuators: Electromechanical modelling and experimental evaluation. Mater. Sci. Eng. C.

[39] Trujillo R., Mou J., Phelan P., Chau D. (2004). Investigation of electrostrictive polymers as actuators for mesoscale devices. Int. J. Adv. Manuf. Technol..

[40] Benslimane M., Kiil H.E., Tryson M.J. (2010). Dielectric electro-active polymer push actuators: Performance and challenges. Polym. Int..

[41] Lau G.K., Lim H.T., Teo J.Y., Chin Y.W. (2014). Lightweight mechanical amplifiers for rolled dielectric elastomer actuators and their integration with bio-inspired wing flappers. Smart Mater. Struct..

[42] Kunze J., Prechtl J., Bruch D., Nalbach S., Motzki P., Seelecke S., Rizello G. (2020). Design and fabrication of silicone-based dielectric elastomer rolled actuators for soft robotic applications. Proc. SPIE.

[43] Shi Y., Askounis E., Plamthottam R., Libby T., Peng Z., Youssef K., Pu J., Pelrine R., Pei Q. (2022). A processable, high-performance dielectric elastomer and multilayering process. Science.

[44] Gilshteyn E., Lin S., Kondrashov V., Kopylova D., Tsapenko A., Anisimov A., Hart J., Zhao X., Nasibulin A. (2018). A One-Step Method of Hydrogel Modification by Single-Walled Carbon Nanotubes for Highly Stretchable and Transparent Electronics. ACS Appl. Mater. Interfaces.

[45] Gilshteyn E., Romanov S., Kopylova D., Savostyanov G., Anisimov A., Glukhova O., Nasibulin A. (2019). Mechanically Tunable Single-Walled Carbon Nanotube Films as a Universal Material for Transparent and Stretchable Electronics. ACS Appl. Mater. Interfaces.

