# Supplementary material for: A Concise Guide to Silicone-Based Spring-Roll Actuator Assembly
Source: Polymers (Basel). 2023 Sep 27;15(19):3908. doi: 10.3390/polym15193908 (PMC10574830; doi:10.3390/polym15193908)
Supplement: Supplementary file 1 [file polymers-15-03908-s001.zip › Supplementary Materials.pdf]

Supporting Information

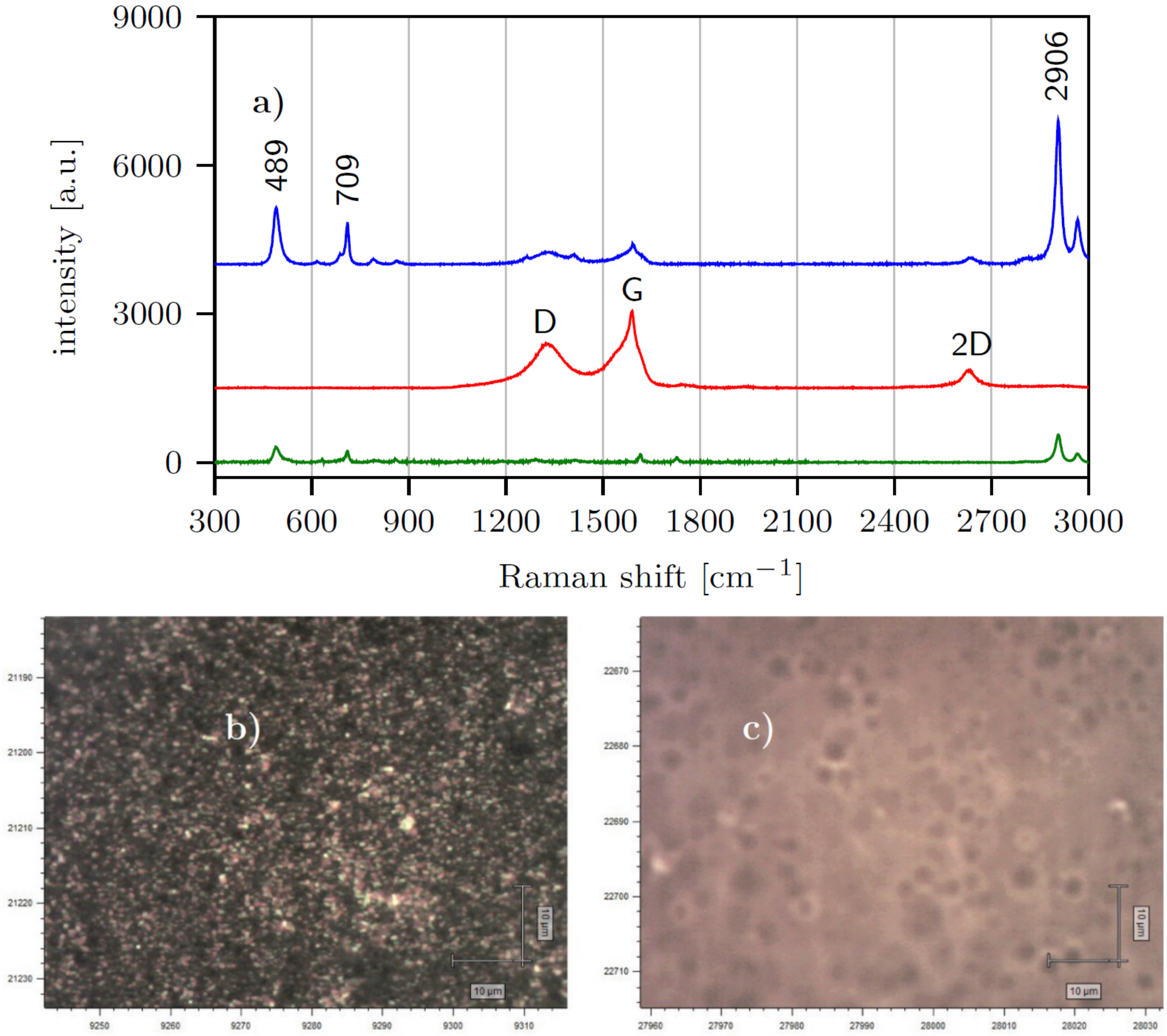

Figure S1. **(a)** Raman spectra of pristine DE film (green), DE film with the CNT/Gr-NH_2 deposited on it (red) and DE film with the CNT/Gr-NH_2 coated with the functional organosilicone polymer composite (blue). The images of the latter two are shown in **(b)** and **(c)**, respectively. Description is presented in the text in detail.

Table S1. Comparison among various rolled DEAs reported so far [37,39–42].

	**Zhang et al [37]**	**Trujillo et al [39]**	**Kunze et al [42]**	**Benslimane et al [40]**	**Lau et al [41]**	**This work**
Dielectric elastomer (DE)	Acrylic VHB 4910	Dow Corning silicone Sylgard 184	Wacker silicone ELASTOSIL 2030	Wacker silicone ELASTOSIL RT625	Silicone BJB TC-5005	Wacker silicone ELASTOSIL 2030
Electrode material	Graphite powder (TIMREX LB1300)	Sputtered gold	Carbon grease	Corrugated silver	Graphite powder (TIMREX KS6)	CNT/graphene
DE roll diameter	12 mm	10 mm	4 mm	—	11 mm	12 mm
DE roll length	45 mm	25 mm	60 mm	100 mm	61 mm	40 mm
Pre-stretch ratio	3×6.5	1×1	1.05×1.05	1×1	1.15×1.15	2×1
Pre-tensioner	Spring	Core free	Core free	Core free	Shell design I	Spring
Pre-tensioner stiffness	200 N m^{-1}	0 N m^{-1}	0 N m^{-1}	0 N m^{-1}	103.0 N m^{-1}	—
Rolled DEA stiffness	1440 N m^{-1}[a]	—	—	3052 N m^{-1}[b]	305.0 N m^{-1}[c]	1500 N m^{-1}
Maximum active strain	31.25 %[c,d]	6.0 %[c]	2.5 %[c]	7.0 %[c]	6.1 %[c]<br>11.0 %[e]	6.0 %[c]
DE breakdown field (E_b)	68.2 MV m^{-1}[f]	50 MV m^{-1}	—	35 – 40 MV m^{-1}	33.5 MV m^{-1}	80 MV m^{-1}
Pre-tensioner weight	7.2 g	—	—	—	1.7 g	4.5 g
Total weight	8.0 g	—	—	—	7.0 g	5.0 g
Static work density	2.25 J kg^{-1}	—	—	—	0.133 J kg^{-1}	1.44 J kg^{-1}
% of max. work density	20.7 %[g]	—	—	—	31.0 %	24.4 %

[a] Calculated using max. blocking force/max. stroke = (7.2 N/5 mm).

[b] Calculated using blocking force/max. stroke = (3 N cm^{-2} × 500 cm × 0.0082 cm)/(6.5 % × 62 mm).

[c] Longitudinal.

[d] Calculated using max. displacement of actuator/active length = (5 mm/16 mm).

[e] Transverse.

[f] Calculated using activation voltage/(initial thickness/(pre-stretch factor)) = (3500 V/(1 mm/3 × 6.5)).

[g] VHB properties: Young's modulus 1.8 MPa, dielectric constant 4.7, density 960 kg m^{-}
